# Editorial

**DOI:** 10.1120/jacmp.v7i2.2308

**Published:** 2006-05-25

Recently, I was impressed with an article authored by Dr. Tim Solberg and published as the Chairman's Report for the ACMP Newsletter. So I asked Dr. Solberg if the JACMP could republish this thought‐provoking article. It is reproduced below, and I hope you will consider his thoughts and the need for change in our profession.

Michael D. Mills, PhD

Would you but a car without looking at it? Would you buy a computer without knowing the price? These seem like ridiculous questions — of course we would never make a major purchase decision unless we clearly understood the quality and price of the item we were about to buy. Yet we are often unwilling, or more aptly unable, to apply the same level of diligence to decisions that literally affect our lives, namely, those involving our health. Our healthcare decisions are largely determined for us, by employers, by insurers, by referral patterns, by personal and business relationships among providers, and by word of mouth from our friends and relatives. Clearly as end consumers we are poorly informed about the quality of our healthcare product and the price which we pay for it. This was borne out in a 2004 survey commissioned by the Henry J. Kaiser Family Foundation, the Agency for Healthcare Research and Quality, and the Harvard School of Public Health, in which 55% of respondents stated that they were currently dissatisfied with the quality of health care. Further, 40% felt the quality of health care had gotten worse over the past five years.^1^ Clearly there is substance to these concerns; as one of many examples, a frequently cited 1999 study from the Institute of Medicine estimated that nearly 100,000 patients die in hospitals each year due to preventable medical errors.^2^ This is more deaths than those due to breast and prostate cancer combined in 2005.^3^

In a 2004 Harvard Business Review Research Report, Michael Porter and Elizabeth Olmsted Teisberg argue that the major challenge facing the practice of medicine in the U.S. is that the nature of competition in healthcare is fundamentally different than in most other industries.^4^ For example, if a particular manufacturer is known to produce faulty television sets, consumers won't buy them. Similarly if the cost of a particular brand of television set is too high relative to its value, consumers won't buy those either. In almost every industry, market forces act to drive up quality while driving simultaneously driving costs down. As a result, TV sets today are both better and cheaper than they were ten years ago. The same can be said for computers and automobiles, cell phone service, fast food, and so on. Market forces are fundamentally different in health care, in large part because neither the quality nor the cost is truly transparent to the consumer. While these arguments may add a different perspective to the dilemma, it comes as a surprise to no one that our nation is facing a crisis in healthcare. We hear the gloom and doom stories on a daily basis. To phrase it in a popular culture term (for those familiar with the Malcolm Gladwell best seller), healthcare is rapidly approaching a “tipping point.”

So what does this have to do with the profession and practice of medical physics? Well I believe that we have an obligation to help to “tip” things in the right direction. This means that we must do all that we can to raise the level of our profession. Specifically, we have an obligation to properly educate and train the next generation of medical physicists. The present standard for medical physics education is through CAMPEP accredited graduate and clinical residency programs. We must take steps to ensure that all practicing medical physicists hold appropriate credentials, and that healthcare providers understand the importance of Board Certified medical physicists in establishing and demonstrating institutional quality standards. We also have an obligation to engage in life‐long learning activities. The standard for medical physics lifelong learning is the maintenance of certification (MOC) process. Finally, we must discourage a one‐size‐fits‐all approach and engage our physics, physician and administration colleagues in embracing evidence‐based medicine. All medicine is not created equal; differentiation based on quality and outcomes will provide much needed transparency and value to the healthcare consumer, transforming the business and the practice of medicine for the better. Our profession has a unique opportunity to take a lead role in shaping these changes.

Timothy D. Solberg, PhD

**Table:** 

The Journal of Applied Clinical Medical Physics	
**Editorial Board 2006**	
Editor‐in‐Chief	Michael Mills
Deputy Editor‐in‐Chief	Timothy Solberg
Associate Editor‐at‐Large	Richard Stark
**Associate Editors**	
Radiation Oncology Physics	Nzhde Agazaryan, Salahuddin Ahmad
	B. Gino Fallone, John Gibbons,
	Michael Herman, Janelle Molloy,
	Matthew Podgorsak, Nikos Papanikolaou,
	Mehrdad Sarfaraz, Lu Wang
Medical Imaging	Geoffrey Clarke, William Pavlicek
Radiation Measurements	Larry DeWerd
Radiation Protection & Regulations	James Deye
Nonionizing Topics	Bhudatt Paliwal
Other Topics	Timothy Solberg
Book/Media Reviews	James Smathers
Editorials	John Horton
**Journal Production Team**	
Proofreader	Heather Shand
Copy Editor	Betty Robinson
Layout Editor	Laura Shand
Manuscript Manager	Patricia Walker
